# Integrating Single-Cell and Spatial Multi-Omics to Decode Plant–Microbe Interactions at Cellular Resolution

**DOI:** 10.3390/microorganisms14020380

**Published:** 2026-02-05

**Authors:** Yaohua Li, Jared Vigil, Rajashree Pradhan, Jie Zhu, Marc Libault

**Affiliations:** 1 Division of Plant Science and Technology, College of Agriculture, Food and Natural Resources, Interdisciplinary Plant Group, Christopher S. Bond Life Sciences Center, University of Missouri-Columbia, Columbia, MO 65211, USA; ylvy9@missouri.edu (Y.L.); jpvxhx@missouri.edu (J.V.); rph93@missouri.edu (R.P.); 2 Division of Biological Sciences, College of Arts and Science, Interdisciplinary Plant Group, University of Missouri-Columbia, Columbia, MO 65211, USA

**Keywords:** plant–microbe interactions, holobiont, microbiome, single-cell RNA sequencing, spatial transcriptomics, multi-omics

## Abstract

Understanding the intimate interactions between plants and their microbiota at the cellular level is essential for unlocking the full potential of plant holobionts in agricultural systems. Traditional bulk and microbial community-level sequencing approaches reveal broad community patterns but fail to resolve how distinct plant cell types interact with or regulate microbial colonization, as well as the diverse antagonistic and synergistic interactions and responses existing between various microbial populations. Recent advances in single-cell and spatial multi-omics have transformed our understanding of plant cell identities as well as gene regulatory programs and their dynamic regulation in response to environmental stresses and plant development. In this review, we highlight the single-cell discoveries that uncover the plant cell-type-specific microbial perception, immune activation, and symbiotic differentiation, particularly in roots, nodules, and leaves. We further discuss how integrating transcriptomic, epigenomic, and spatial data can reconstruct multilayered interaction networks that connect plant cell-type-specific regulatory states with microbial spatial niches and inter-kingdom signaling (e.g., ligand–receptor and metabolite exchange), providing a foundation for developing new strategies to engineer crop–microbiome interactions to support sustainable agriculture. We conclude by outlining key methodological challenges and future research priorities that point toward building a fully integrated cellular interactome of the plant holobiont.

## 1. Introduction

Plants are increasingly recognized as holobionts, comprising the host and its symbiotic microbiome in interaction [1]. Soil and plant microbiomes play critical roles in plant development, immunity, and environmental responses by contributing to nutrient cycling, disease resistance, and stress tolerance [2,3,4,5]. Over the past decade, metagenomic and amplicon-based studies have greatly expanded our knowledge of microbial community composition and ecological dynamics in diverse agricultural systems [6,7,8,9]. However, these community-level surveys often overlook the spatial distribution and functional specificity of plant–microbe interactions, such as how individual plant cell types perceive, interpret, and respond to microbial cues in a developmentally regulated context [10,11].

Advances in single-cell and spatial multi-omics now offer unprecedented opportunities to bridge this gap. Techniques such as single-cell RNA sequencing (scRNA-seq), single-nucleus RNA sequencing (snRNA-seq), and spatial transcriptomics have uncovered profound cellular heterogeneity in plant organs [12,13]. For instance, in legumes, scRNA-seq revealed distinct transcriptional profiles between infected and uninfected cells within nodules, mapped the zonation of fixation in indeterminate nodules and the cellular heterogeneity in the infection zone of determinate nodules, and highlighted metabolic and developmental compartmentalization (e.g., ureide biosynthesis/transport programs), cytokinin-associated regulatory modules, and transitional infected states enriched for nodulation and infection-thread-related genes with remarkable resolution [14,15,16,17]. Equally important, recent single-cell and spatial transcriptomics in plant–microbe symbioses extend these insights beyond nodulation to arbuscular mycorrhizal (AM) interactions, establishing transcriptional programs in a subset of root cortical cells, and their regulation with respect to colonization stage and spatial position within the root [18,19]. Spatial transcriptomic profiling further shows that fungal gene expression can be mapped in situ alongside host transcripts, although transcriptional specialization within microbial populations often remains difficult to resolve due to technical constraints in microbial RNA capture. Together, these datasets support the view that cell-type-specific host programs are matched by spatially coordinated microbial activities, reinforcing the concept of tightly synchronized cellular dialogs within the holobiont. Similarly, single-cell studies in *Arabidopsis thaliana* have provided complementary insights into plant–pathogen interactions, identifying cell-type-specific immune signatures and spatially resolved defense networks during microbial infection [20,21]. These data suggest that plant responses to microbes are orchestrated not only at the tissue level, but also spatially at the cellular level, where spatial organization, hormonal gradients, and cell fate converge to mediate colonization, defense, or exclusion.

Single-cell studies also reveal that host immune architecture varies across tissues. In maize, for instance, bundle sheath and guard cells exhibit pre-activated immune states that likely enable rapid responses to pathogens [22,23]. Time-resolved single-cell and single-nucleus analyses in *A. thaliana* have further highlighted that immune responses differ across leaf cell types and over time. Notably, circadian regulation contributes to these dynamics [20]. These findings underscore the limitations of bulk-level measurements from heterogeneous tissues and motivate a shift toward dissecting immunity and symbiosis at single-cell resolution.

To further understand host–microbe interactions, integrative multi-omics approaches that combine transcriptomics with assays of chromatin accessibility (e.g., ATAC-seq) and histone modifications (e.g., ChIP-seq), together with metabolomics, are crucial yet remain largely underexplored to decipher plant–microbial systems [8,12,19,24]. Such approaches will help identify transcriptional regulators and metabolic zones but will also enable functional inference of regulatory circuits that control microbial recruitment and host reprogramming. At the same time, they expose important technical and conceptual limitations, including low efficiency of microbial RNA capture, high background from plant ribosomal RNAs and underdeveloped pipelines for cross-species and cross-kingdom annotation, particularly in non-model crops.

In this review, we summarize emerging insights from single-cell and spatial multi-omics on the dynamic and cell-type-specific interactions between plants and microbes. We highlight how these approaches illuminate developmental, immunological, and symbiotic processes at cellular resolution and critically assess their current limitations. Finally, we discuss future directions for translating single-cell and spatial insights into microbe-informed strategies for sustainable agriculture while cautioning against overly simplistic applications that ignore ecological context, developmental stage, and environmental variability.

## 2. Plant Cell-Type-Resolved Immune and Symbiotic Responses

### 2.1. Legume Host Cell States in Symbiosis

Legume nodulation is initiated by the infection of root hair cells by rhizobia and the initiation of cell divisions in the cortical cell layer of the root. Using the *Medicago truncatula* nodule primordia as a model, Pereira et al. [25] established the trajectory inference starting from the root cortical cells and ending with the formation of the nodule meristem. Following the establishment of this meristem, the Medicago indeterminate nodule develops. When mature, the indeterminate nodule is characterized by the presence of a permanent meristem and a zonation of the cells engaged in a symbiotic relationship with rhizobia: (I) the division zone, (II) the infection zone, (III) the nitrogen fixation zone, and (IV) the senescence zone. In contrast, determinate nodules (e.g., the soybean nodule) do not maintain a permanent meristem and the cells infected by rhizobia are not organized in specific zones [26]. However, they are composed of different states of rhizobia-infected cells: (I) those engaged in endoreduplication, (II) the cells actively fixing nitrogen, and (III) cells engaged in senescence [17]. Regardless of nodule determinacy, inferring transcriptomic trajectories across nodule zones and host cell states is required to reveal the genetic programs governing plant–rhizobia symbiosis and their regulation.

### 2.2. The Advent of Bacterial Single-Cell Technologies

Recent advances in single-cell transcriptomic technologies now enable bacterial transcriptomes to be profiled at the single-cell resolution. Until recently, bacterial single-cell transcriptomics were largely inaccessible because of several technical barriers [27] including their very low RNA amounts (i.e., estimated to be up to ~100-fold lower than in eukaryotic cells), and much shorter RNA half-lives compared to eukaryotic RNA, the consequence of the absence of a 5′ cap [28] and 3′ polyadenylated tail [27]. In addition, many popular single-cell chemistries use the eukaryotic poly(A) tail to initiate cDNA biosynthesis, a feature often absent from bacterial transcripts. Finally, bacterial cell wall permeabilization and lysis must be rapid and efficient while minimizing RNA degradation.

Upon overcoming all these limitations, single-cell sequencing of bacteria can reveal transcriptionally distinct microbial subpopulations that are obscured in bulk analyses. Although bacteria reproduce asexually, individual cells can occupy distinct phenotypic states and respond heterogeneously to the same stimulus [29]. Nevertheless, these heterogeneous cells can still coordinate collective behaviors at the community level [30]. Indeed, bacteria can engage in diverse cooperative programs that promote colony persistence such as horizontal gene transfer, in which cells exchange genetic material to diversify their genetic pool [31]. Other tasks like quorum sensing, bet hedging, or biofilm formation require population signaling and are more complex [30,32]. To date, bacterial single-cell sequencing has only been applied to strains relevant to human pathology, such as *Escherichia coli* or *Salmonella enterica* [32,33]. Taken together, the emergence of bacterial single-cell approaches creates a timely opportunity to extend single-cell frameworks to plant-associated symbionts and, importantly, to interrogate plant–microbe interactions in the context of the multi-partner holobiont network.

### 2.3. Understanding the Complex Holobiont Network and Its Interactions

Despite recent advances that make bacterial single-cell transcriptomics technically feasible, most applications in plant systems remain limited to simplified or controlled settings. Microbial single-cell profiling is most achievable when the microbial component is experimentally tractable. For example, in single strains or low-complexity plant-associated communities, experimental handling (including permeabilization/lysis) and read assignment are generally more manageable, especially when reference genomes are available [27,33]. In contrast, applying these approaches to complex, soil-derived holobiont communities remains largely aspirational. In field soils, key barriers include low microbial biomass, extreme community diversity, incomplete reference genomes, and the presence of closely related strains, all of which complicate demultiplexing and reliable taxonomic assignment of transcripts. Moreover, many soil bacterial taxa remain undescribed and are not readily culturable, further limiting annotation quality and downstream interpretation [34,35]. Together, these constraints highlight that while microbial single-cell profiling is technically within reach for controlled plant-associated systems, substantial experimental and computational advances are still required for robust single-cell and spatial profiling in complex field soil communities.

As a holobiont, there are many different microbes interacting with each other at once and with plants [36]. These interactions can have positive, negative, or neutral effects on microbial fitness and on the host plant. In *A. thaliana*, infection from *Pseudomonas syringae* DC3000 combined with scRNA-seq and pseudotime analysis allowed researchers to resolve plant cell-type-specific responses and their associated transcriptomic trajectories [37]. Interestingly, the defense responses of the epidermal, mesophyll, and vascular cells followed two alternative trajectory branches before converging on a common response program. This may highlight the difference between cells physically interacting with the pathogen and those engaged in a systemic response. These interactions also influence the cellular composition of the holobiont [38]. For instance, microbes may compete with one another for host-derived resources. Some *Pseudomonas* species produce the secondary metabolite 2,4-diacetylphloroglucinol [4] known for its antibacterial and antifungal properties. As another example, in a synergistic instance, it has been found that some bacteria form biofilms to attach to the hyphae of AMF [38] to benefit from hyphal exudates [39]. They may also benefit from dispersal along hyphal networks into new soil niches, while contributing to organic phosphorus mineralization and thereby supporting AM-mediated phosphorus delivery to the host plant. Together, these studies indicate that holobiont outcomes are governed by spatiotemporally structured interactions and cannot be inferred from community composition alone.

### 2.4. Symbiosis Within the Holobiont

In many systems, the host actively modulates symbiotic outcomes through constraints that limit costs and stabilize mutualism. In legumes, for example, excess nitrogen availability can trigger nodule senescence and terminate the association, in some cases leading to bacteroid death [26]. In the case of AMF, arbuscules are short-lived and degenerate after two to three days [40]. This rapid turnover has been proposed as a host-level safeguard that limits fungal exploitation when phosphorus delivery is insufficient relative to carbon investment. With arbuscules constantly being formed and deteriorating every two to three days, multiple developmental stages of arbuscules can coexist within the cortex simultaneously [18]. This arbuscular developmental dynamic adds another aspect to consider when performing transcriptomic studies on AM symbiosis. Accordingly, whole-root or bulk analyses may mask the transcriptomic complexity of coexisting arbuscule states and obscure stage-specific programs. AMFs are experimentally challenging because they are obligate biotrophs, cannot be readily cultured and do not complete their life cycle without symbiotic conditions. As a result, the plant and AMF transcriptomic mechanisms that support this symbiosis have been difficult to resolve. Recently, using snRNA-seq and spatial transcriptomics, researchers have co-captured plant and fungal transcriptomes in the symbiosis between *M. truncatula* and *Rhizophagus irregularis* [18]. In this study, transcriptomic differences between AMF-colonized cortical cells and uncolonized cells were resolved. They were also able to distinguish between different phases of arbuscule development.

**Figure 1:**
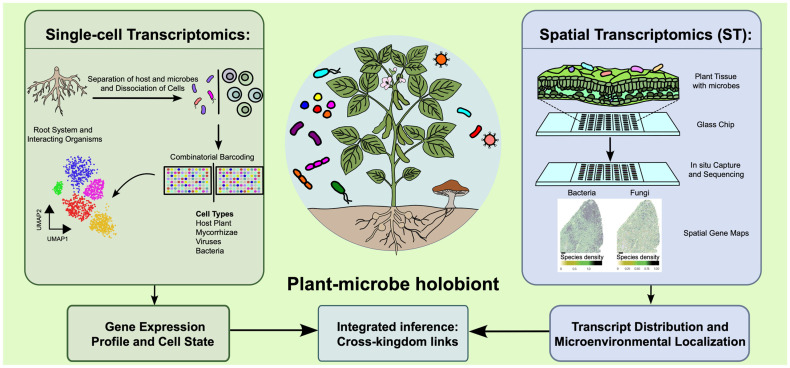
Deciphering the plant holobiont through single-cell and spatial transcriptomics. In the center is a photo of a host plant and the many organisms it will interact with in the holobiont. The left side depicts the general workflow for scRNA-seq of the host plant and the microbes of the plant holobiont. The right side shows a general workflow for spatial transcriptomic methodologies. Combined, these approaches will dissect the interactions between the host plant and its microbes. The spatial gene expression map of a leaf is from Saarenpää et al. [41].

The ability to analyze multiple different transcriptomes simultaneously (i.e., plant and associated microbes) represents an important step towards understanding the complexity of the holobiont (Figure 1). As multi-kingdom profiling technologies advance, holobiont interactions can be reframed from simplified conceptual models to empirically grounded, network-level mechanisms supported by cell- and state-resolved data.

## 3. Spatial Signatures of Plant–Microbe Interactions

Plant–microbe interactions are shaped by both the composition of the microbial community and the tissue microenvironment [41]. Unlike animals, with mobile immune cells, plants depend on stationary cells to mount localized responses and coordinate signaling across tissues [42]. Consequently, spatial context within the tissue microenvironment is critical for balancing effective immunity with growth and symbiosis. Recent advances in single-cell and spatial transcriptomics have enabled a deeper understanding of how plants orchestrate spatially organized cellular responses to microbial interactions.

### 3.1. Spatial Zonation in Plant Immune Responses

Plants exhibit distinct spatial zonation in their immune responses to microbial infection, creating specialized microenvironments that optimize defense strategies [43,44,45,46,47,48]. For instance, in Arabidopsis roots, immune marker genes are strongly induced in the elongation zone but show little or no expression in adjacent regions in response to microbe-associated molecular patterns (MAMPs) treatment [43,44]. However, these unresponsive zones can gain immune competence in the presence of damage signals, resulting in spatially restricted defense activation [44,45]. Similar spatial patterns occur in leaves, where immune gene expression is highly induced around infection sites [21,41,47,49,50,51,52]. This spatially restricted defense is compartmentalized across different cell layers, with distinct cell states exhibiting varying immune intensities [21,47]. The spatial distribution of induced immune genes correlates with immune receptor expression in specific cells or cell types. For example, the pattern recognition receptor Flagellin Sensing 2 (FLS2) is preferentially induced in cells or tissues susceptible to microbial entry, such as stomata, hydathodes, and lateral roots [53]. Recent single-cell and spatial transcriptomic studies have further revealed cell-type-specific immune programs during microbial colonization, emphasizing the fine-scale coordination of plant defense across diverse spatial contexts [21,54,55]. Together, these findings suggest that plants selectively activate immunity in vulnerable zones while minimizing fitness costs.

### 3.2. Spatial Zonation in Plant–Microbe Symbioses

Plant–microbe symbioses, similarly, display spatial zonation of host responses, reflecting specialized cellular functions during symbiosis establishment and maintenance. This is well-exemplified in legume–rhizobium interactions. As mentioned above, in indeterminate nodules, host cells and rhizobia are spatially arranged along a longitudinal developmental axis into defined meristematic, infection, differentiation, nitrogen-fixing, and senescence zones, each with unique transcriptional and metabolic profiles [56,57,58]. By contrast, determinate nodules lack this longitudinal zonation; instead, host cells and rhizobia are organized around a central infection zone where infected and uninfected cells co-exist [15,17]. Within the infected cell population of determinate nodules, Cervantes-Perez et al. [17] further resolved two different subpopulations: those engaged in endoreduplication and intense transcriptional activity, and those involved in active nitrogen fixation.

At the biochemical level, single-nucleus transcriptomic studies in *M. truncatula* revealed that flavonoid synthase genes essential for rhizobia recruitment are selectively expressed in cortical cells, but not in neighboring tissues [16]. Further work reveals that flavonoid biosynthesis is most pronounced in the elongation zone of the root [59]. These findings underscore that the root microenvironment is heterogeneous, and symbiotic activity is localized to discrete domains. Spatial compartmentalization enables plants to precisely regulate symbiotic activity, supporting efficient nutrient allocation while limiting access to infected or non-beneficial zones, and reinforcing mutualism stability [48,60,61]. It also provides developmental flexibility, allowing for localized symbiont accommodation without disrupting overall root function or immunity [62,63,64]. Advances in single-cell and spatial omics now allow for high-resolution mapping of these zones, revealing new insights into cell-type-specific signaling, nutrient exchange, and immune regulation [11,16,48,57,58,65]. These insights open avenues for enhancing symbiotic efficiency by targeting competent cell populations or modulating zonal transitions.

These spatial zonation patterns reflect cell layer- and cell type-specific response capacities, suggesting that not all cells contribute equally to local infections. Instead, plant responses are spatially restricted and fine-tuned according to cellular identity and proximity to microbial signals. This challenges the notion of uniform immune competence across plant cells, unveiling how plants create specialized microenvironments that serve different functions in the overall interaction strategy. In summary, spatial zonation is a fundamental feature of plant–microbe interactions, enabling specialized microenvironments for infection, signaling, and nutrient exchange. Such organization ensures that interactions are both efficient and tightly regulated, maintaining balance between microbial engagement and overall tissue function and development.

## 4. Integrating Multi-Omics to Reconstruct Interaction Networks

While single-omic profiles reveal cell states, interaction mechanisms often require linking transcription, chromatin regulation, and metabolic outputs across space. In this section, we discuss integrative multi-omics strategies that connect sc/snRNA-seq with single cell ATAC-seq, spatial transcriptomics, and metabolomics to infer causal regulators and interaction niches. We emphasize analytical considerations specific to plant–microbe systems, including cross-kingdom feature annotation, compositionality, and the need for benchmarking with perturbation and imaging-based validation. As a roadmap for the integration strategies discussed below, Table 1 compares the major approaches and their typical trade-offs.

### 4.1. Single-Cell Multi-Omics Reveals Regulatory Complexity in Plant–Microbe Interactions

Cell biology is governed by multiple, interconnected layers of molecular regulation. In systems that involve a symbiotic partner such as root nodules, AM symbiosis, or plant–pathogen interactions, this regulatory complexity increases further, as cellular programs are now modulated by an external biotic factor. Importantly, the regulation of these programs is bidirectional: the plant reshapes microbial behavior, and the microbe actively modulates plant regulatory networks. Single-cell transcriptomic approaches have been instrumental in establishing cell-resolved gene expression atlases of holobionts [48,73]. However, we must recognize that transcriptomes alone are insufficient to fully reveal regulatory mechanisms or the full extent of cellular heterogeneity. The integration of various single-cell and spatially resolved datasets (e.g., scRNA-seq with complementary spatial transcriptomic approaches, scATAC-seq to map accessible chromatin, ChIP-seq to identify transcription factor binding-motifs and histone modifications, and spatially resolved metabolomic datasets to connect regulatory and gene expression changes to metabolic function [74]) would enable a far more comprehensive dissection of cell-type-specific regulatory responses that shape plant–microbe symbioses.

### 4.2. Reconstructing Cell-Type-Specific Regulatory Networks via scRNA-seq and scATAC-seq Integration

Co-capture from the same nuclei of scRNA-seq and scATAC-seq profiles enables direct inference of TF-target relationships by linking motif-containing accessible chromatin regions to changes in gene expression within the same cell populations. This approach leads to more accurate reconstruction of gene regulatory networks than using expression data alone. Farmer et al. [69] and Dorrity et al. [66,69] applied scATAC-seq to *A. thaliana* roots and demonstrated that chromatin accessibility patterns and transcriptomes each provide independent definitions of cell identity [66,69]. By integrating single-cell chromatin accessibility with gene-expression profiles, Dorrity et al. [66,69] reconstructed developmental trajectories including transitions associated with endoreduplication and cell-division states. This multi-omics framework also identified TF families enriched in specific cell types and linked changes in TF expression to corresponding shifts in chromatin accessibility at their target loci, helping distinguish directly from indirect regulatory influences. Similarly, Farmer et al. [69] used a combinatorial approach of snRNA-seq and single-nuclei ATAC-seq and showed that differential chromatin accessibility is a key mechanism underlying cell-type-specific gene regulation.

At the bulk resolution, integration of ChIP-seq and RNA-seq in several studies has uncovered transcriptional reprogramming driven by changes in histone modifications. For example, in soybean nodules, H3K4me3 levels positively correlated with gene expression, and reduced H3K4me3 marking at several TF loci corresponded with repression of defense-related pathways [67]. Similarly, combined ChIP-seq and RNA-seq analyses in *Paulownia fortunei* revealed that phytoplasma-induced transcriptional reprogramming is partly mediated by changes in activating histone marks [75]. Considering the dynamic nature of the response of plant cells to microbial infection, the application of scRNA/ATAC-seq integrated approach and its extension to other dimensions such as ChIP-seq would likely be transformative to our understanding of plant cell immunity of during microbial infections.

### 4.3. Advances in Single-Cell and Spatial Metabolomics for Plant Biology

Single-cell mass spectrometry (MS) faces challenges like the low abundance of analytes and the small volume of individual cells (femtoliter to nanoliter range). Despite this, significant progress has been made in developing sensitive and robust single-cell MS approaches [72]. However, only a few methods have been successfully adapted for plant cells. To date, most analyses in plants have relied on mass spectrometry imaging (MSI) or live single-cell mass spectrometry (LSC-MS) [76,77,78,79]. For instance, ultra-high-resolution (FTICR) mass spectrometry imaging has been applied to poplar leaves under water deficit stress, revealing unique metabolomes of various cell types [80]. Recently, integrating spatially resolved gene expression and metabolomic profiles in soybean seeds has opened new avenues for understanding plant–microbe interactions [81]. Similarly, Li et al. [82] developed a high-throughput, high-resolution, semi-quantitative single-cell metabolomics method for leaf cells. This method complements their findings from single-cell transcriptomics, which uncovered a 38-step monoterpene indole alkaloid (MIA) biosynthetic pathway across three distinct cell types in *Catharanthus roseus* leaves [82]. Their approach used a precision microfluidic robot to isolate protoplasts from leaves prepared with a SIEVEWELL device. These protoplasts were then transferred to 96-well plates compatible with an ultra-high-performance liquid chromatography-mass spectrometry (UPLC-MS) autosampler. This advanced technique identified metabolites that co-occurred in different cell types and quantified metabolite concentrations across the cell population.

Together, these advances highlight the growing importance of spatial metabolomics, which integrates MSI and metabolomics for in situ identification, localization, and quantification of metabolites within tissues and cells. While spatial metabolomics is widely used in medicine and animal research, its application in plant biology is still developing [71]. Accurate mapping of metabolite distributions is crucial, as plant metabolite biosynthesis is highly spatially organized and linked to physiological functions. For example, in legume root nodules, symbiotic establishment and nitrogen fixation depend on organized spatial and metabolic structures across different compartments such as infection zone, nitrogen-fixing zone, and vascular tissues. When integrated with transcriptomic analyses, spatial metabolomics can help identify metabolic niches that support microbial accommodation, regulate nutrient exchange, and enhance our understanding of the molecular and metabolic networks in nodule development and legume–rhizobia symbiosis.

### 4.4. Connecting Transcriptomes to Metabolomes at Single-Cell Resolution

Integrating single-cell metabolomics with scRNA-seq can enhance our understanding of cellular heterogeneity in plants. While scRNA-seq provides high-resolution information on gene expression in individual cells, it does not capture the functional output of gene regulation: the metabolome. Single-cell metabolomics measures metabolites directly within cells, but it lacks insights into the regulatory programs governing their production [83]. By capturing both modalities from the same cells, researchers can correlate gene expression with metabolite levels, revealing how transcriptional states influence metabolic phenotypes [84]. The recent study in *C. roseus* found that gene expression did not always match metabolite levels within the same cell type. This discrepancy helped identify candidate biosynthetic enzymes and transporters through gene-metabolite correlation analysis [82]. Integrated single-cell multi-omics allows for precise mapping of metabolically specialized cells. It elucidates cell-to-cell variability in metabolic states and provides a powerful tool for linking genotype to phenotype at an unprecedented resolution.

## 5. Future Perspectives and Challenges

### 5.1. Technical Limitations in Single-Cell Plant–Microbe Studies

Current single-cell and spatial transcriptomic frameworks remain essentially plant-centric. Standard single-cell chemistries are designed for eukaryotic cells and nuclei, not for microbial populations [68,85,86,87,88]. Plant and bacteria both possess cell walls, a major limitation when applying single-cell technologies. To overcome this limitation, plant biologists digest the plant cell wall (i.e., protoplastization) or isolate nuclei to use them as biological entities for single-cell RNA-seq and ATAC-seq analyses. Such a solution cannot be directly applied to bacteria. In addition, incomplete reference genomes for plant-associated microbes, together with strain-level diversity and horizontal gene transfer, make it difficult to quantify microbial load or resolve strain-specific functions at single-cell resolution. Future method development therefore cannot be a simple incremental extension of animal or purely plant pipelines. It must explicitly address microbial constraints (i.e., capture chemistries that can recover non-poly(A), low-abundance transcripts) towards the development of new single-cell strategies that reveal the biology of individual microbes, and/or retain microbe–host cell interactions. In parallel, new analysis frameworks are needed that explicitly model uncertainty in cross-kingdom mapping, rather than discarding or collapsing microbial reads as technical noise.

### 5.2. Standardizing Multi-Omics Integration and Developing Microbe-Compatible Spatial Methods

Plant single-cell research is beginning to adopt general multi-omics integration tools, but practices remain fragmented and most methods have been benchmarked primarily on animal and human data [89,90]. Different plant single-cell studies apply heterogeneous normalization, batch correction, and clustering strategies, making it difficult to compare atlases or to reuse them as references [68,86]. In plant–microbe systems, these issues are even more acute: naively co-embedding host and microbial features in a single latent space can downweight or obscure microbial variation, because host and microbiota differ markedly in genome architecture, gene density, and expression ranges [3]. The lack of widely adopted minimal reporting standards for inoculum composition, colonization level and spatial resolution further prevents systematic cross-study synthesis.

Methodologically, we still lack integration frameworks that are explicitly designed for cross-kingdom data. Future pipelines will likely need to maintain partially separate latent spaces for host and microbes, allow for asymmetric normalization, and connect the two compartments through biologically interpretable links such as metabolite exchange, ligand–receptor pairs or co-regulated gene modules. On the experimental side, spatial transcriptomics in plants has so far been applied mainly to host gene expression, with bacterial and fungal partners remaining largely invisible [70]. Although recent work in soybean nodules represents an early step toward cross-kingdom spatial mapping—by using Molecular Cartography to detect a small panel of rhizobial transcripts alongside host gene expression—microbial transcript coverage remains limited, underscoring the need for both improved capture chemistry and microbe-aware integration models [17]. Adapting high-plex in situ strategies, such as MERFISH, Xenium or HCR-FISH-like approaches, to the optical and structural constraints of plant tissues, and coupling these measurements to microbe-aware integration models, should therefore be a priority if we want spatial maps that truly describe both sides of the interaction rather than only the plant.

### 5.3. Mapping Plant–Microbe Interactions Across Developmental Stages and Environmental Contexts

Current datasets provide exquisitely detailed yet fundamentally static snapshots of plant–microbe interactions, which limits our ability to resolve inherently microbial dynamics such as recruitment, establishment, persistence, and functional state switching. A major driver of temporal variation is host development, which reshapes immune thresholds and symbiotic competence even within the same organ [86,91]. At the community level, longitudinal field studies demonstrate that plant developmental stage can explain as much or more variance in microbiome composition, ecological networks and functional roles as soil type or location, with the maize microbiome being a prominent example [3,92]. To date, apart from the development of pseudotemporal transcriptomic trajectories, single-cell atlases have not captured these temporal reorganizations.

Therefore, future research should map dynamic plant–microbe interactions across developmental stages and environmental conditions, including combinations of abiotic stresses, with explicit readouts of microbial colonization and function over time. Increasing evidence shows that both immune and symbiotic responses are tightly coordinated with developmental programs. For example, key immune receptors such as the cold shock protein receptor (CORE) and FLS2 exhibit developmentally regulated expression, suggesting that receptor deployment is aligned with developmental stage [93,94]. Development also shapes the assembly and activation of downstream gene modules [95]. These modules, comprising TF and signaling components, are often controlled in a development-dependent manner [96]. Environmental factors, such as drought, salinity, heat, and nutrient limitation, further modulate plant–microbe interactions by rewiring signaling cascades, altering microbial behavior and community composition in ways that feedback on host signaling [97,98,99,100]. In conclusion, plants must decode and integrate overlapping microbial, environmental and developmental signals to fine-tune their responses, often using shared signaling components [101,102]. How plants diversify outputs from these shared pathways, producing distinct outcomes under different environmental and developmental conditions, remains an open question. Collectively, spatiotemporal mapping of plant–microbe interactions across developmental trajectories and environmental gradients will provide critical insights into how plants coordinate defense, symbiosis, and growth. Such efforts will be essential for improving plant resilience in dynamic and often hostile environments.

### 5.4. Applications in Sustainable Agriculture: Precision Inoculant Design and Host Trait Engineering

Microbiome-based products are often promoted as an obvious route to “sustainable agriculture”, yet field performance of microbial inoculants can vary across environments [3,103]. Nevertheless, recent research on microbiome-based solutions and microbiome engineering shows that both synthetic communities (SynComs) and natural communities (NatComs) can deliver measurable benefits that are reproducible under certain field conditions [104,105,106]. Despite these successes, many consortia and strains are still selected empirically, with limited mechanistic understanding of which host cell types they colonize, which microbial subpopulations drive beneficial functions, or under which environmental regimes they remain effective. Single-cell and spatial omics have the potential to transform this situation by precisely depicting the response of plant cells to microbial inoculations. In principle, such datasets can identify permissive versus restrictive host cell states, pinpoint receptors, TFs and metabolic programs that correlate with successful colonization, and resolve functional heterogeneity within microbial populations that is invisible in bulk measurements [85,86,103]. Upon implementing such a strategy, single-cell data will become a target-discovery layer for precision inoculant design and cell-type-specific host engineering, rather than an end in themselves.

Importantly, engineering host traits or designing “elite” consortia based solely on controlled-condition single-cell datasets might ignore the strong context dependence revealed by developmental, environmental and community-level studies. For instance, the ecological role and net effect of the same microbial taxon can shift with plant age, soil background and stress combinations [92,99,100]. A more realistic roadmap is stepwise and explicitly iterative. First, single-cell and spatial data can be used in simplified systems (e.g., gnotobiotic setups or controlled greenhouse experiments) to nominate candidate host regulators and microbial strains or subpopulations that are associated with robust colonization or desirable host responses. Second, these candidates should be tested across contrasting host genotypes, soil types and stress regimes in mesocosm and field trials to evaluate their performance, stability and ecological side effects. Third, the results of these trials should feed back into the single-cell and spatial analyses, allowing researchers to iteratively refine both host targets and inoculant composition based on agronomic performance, ecological impact and robustness. In this view, single-cell and spatial multi-omics do not magically “solve” sustainable agriculture. What they can do is sharpen the questions we ask and narrow the design space for breeding and microbiome engineering. To realize this potential, they must be treated as one component of an experimental cycle that runs from mechanistic discovery through multi-environment testing to agronomic outcomes, rather than as self-sufficient endpoints.

## Figures and Tables

**Table 1:** Comparison of single-cell and spatial omics methods for plant–microbe studies.

Method	Primary Readout	Sample/Prep Requirement	Spatial Resolution	Sensitivity/Capture Efficiency	Throughput	Best-Fit Biological Questions	Key Strengths	Key Limitations/Downsides (Explicit Trade-Offs)
scATAC-seq [66]	Chromatin accessibility per cell/nucleus	Usually, nuclei	None	Low–medium (sparse peaks)	High	Regulatory programs and TF motif activity underlying plant states; priming under stress/infection	Adds regulatory layer; integrates with RNA	Accessibility ≠ activity; sparse; prep-sensitive; interpretation requires integration
ChIP-seq/CUT&Tag/CUT&RUN [67]	TF binding/histone marks	Crosslinking or enzyme-tethering	None	Medium–high (target-dependent)	Low–medium	Validate regulatory mechanisms for plant responses (specific factors/marks)	Mechanistic regulatory evidence	Antibody dependence; often population-average; cost/complexity
Bulk RNA-seq [3]	Tissue-average gene expression	Bulk tissue RNA	None	High	High (samples)	Global plant response; pathway-level DE; treatment comparisons	Mature, cost-effective; high DE power	Masks heterogeneity; plant-dominant signal can obscure microbial transcripts; cannot link to cell types or niches
scRNA-seq [68]	Single-cell gene expression	Single-cell isolation	None	Medium (dropouts common)	High (cells)	Plant cell-type atlas; rare plant states; transcriptional programs under infection/stress	Highest plant cellular resolution; identifies rare cell types	Dissociation stress and cell-type bias; hard-to-dissociate tissues; costly; sparse matrices
snRNA-seq [69]	Nuclear RNA (incl. pre-mRNA) per nucleus	Nuclei isolation	None	Medium–low (nuclear bias)	High (nuclei)	Plant atlas when protoplasting is biased/impossible; developmental gradients; cell-state changes in rigid tissues	Works with frozen tissue; reduces dissociation artifacts	Lower sensitivity for cytosolic/low-abundance genes; pre-mRNA complicates quantification; still no spatial context
Sequencing-based spatial transcriptomics [70]	Spatially indexed plant transcripts	Tissue section + capture on slide	Medium (platform-dependent)	Medium–low (platform-dependent)	Medium (spots)	Where plant programs occur in tissue; spatial domains/niches (plant-defined); microenvironmental heterogeneity	Preserves architecture; links expression to location; integrates with histology	Trade-off: spatial context ↑ but capture efficiency/sensitivity often ↓; resolution may be below single cell; analysis complexity; cost
Image-based spatial transcriptomics [24]	Targeted transcripts with cellular/subcellular localization	Tissue section + probe design for selected genes	High (single cell/subcell)	Medium–high for targeted genes	Medium	Microbe localization; plant–microbe co-localization; niche mapping; marker-based interaction hypotheses	True spatial localization; interpretable; compatible with morphology	Trade-off: spatial resolution ↑↑ but transcriptome breadth ↓ (targeted panels); probe design burden; optical constraints in plant tissues
MSI/Spatial metabolomics [71]	Spatial metabolites/lipids/chemicals	Tissue section + instrument-specific prep	Medium (platform-dependent)	Low–medium (rare metabolites hard)	Medium–low	Metabolic niches; chemical gradients around infection/symbiosis; functional microenvironments	Functional molecules; broad chemistry; label-free	Quantification/ID ambiguity; cell/taxon attribution is indirect; cost/technical complexity
Single-cell mass spectrometry (SCMS) [72]	Metabolites/lipids/proteins per single cell	Isolated single cells	None	Low (rare analytes)	Low	Direct functional molecules at single-cell level	Direct chemistry; label-free	Very low throughput; destructive; limited coverage; plant cell isolation challenges
Microbial single-cell transcriptomics [27]	Microbial gene expression per cell	Microbe enrichment/isolation	None	Low–medium (tiny RNA; capture hard)	Low–medium	Microbial heterogeneity; state switching within a strain/consortium (when feasible)	Direct microbial state readout	Low RNA content; biases; community complexity; strain-level variation; mapping/annotation uncertainty

Note: ↑ indicates increased, ↓ indicates decreased, and ↑↑ indicates substantially increased.

## Data Availability

No new data were created or analyzed in this study. Data sharing is not applicable to this article.
